# Obituary

**DOI:** 10.1186/s12948-018-0096-5

**Published:** 2018-08-07


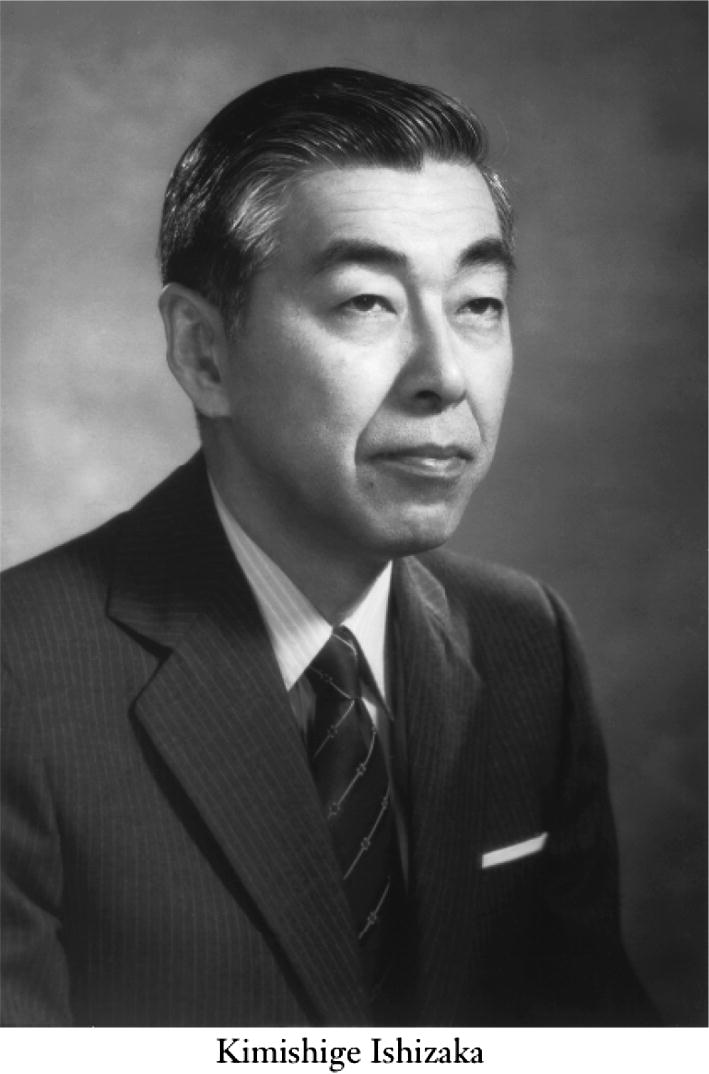


On July 6, 2018, at the age of 92, Professor Kimishige Ishizaka has died. Prof. Ishizaka, known as Kimi from his friends and colleagues, together with his wife Teruko, known as Terry, discovered IgE antibodies in 1966. Kimi Ishizaka was born on December 3, 1925, graduated in Medicine in 1948 and obtained his Ph.D. in 1954 at the University of Tokyo, quickly became Chief of the Department of Serology at the National Institute of Health in Japan. From 1957 to 1959 he was a Research Fellow at the California Institute of Technology (CALTECH) in Pasadena under the enlightened guidance of Prof. Dan H. Campbell. From 1962 to 1970 he was Chief of the Department of Immunology at the Children’s Asthma Research Institute at the University of Colorado Medical School. This last period was of fundamental importance in the scientific career of Kimi and Terry Ishizaka who, in 1966, using a series of sophisticated techniques, showed unequivocally the existence in the serum of allergic patients of a new class of immunoglobulins, which was named IgE, later recognized by the WHO. This extraordinary discovery has allowed the development of modern Allergology.

The move of the Ishizakas in 1970 to Johns Hopkins University in Baltimore marked a turning point in their scientific evolution. For almost 30 years an unrepeatable scientific collaboration was realized between the Ishizakas, Basic Immunologists, and the group of Philip Norman and Lawrence M. Lichtenstein, Clinical Immunologists and Allergists. This fantastic scientific interaction was also possible thanks to the proximity of the Divisions of Basic Immunology and Clinical Immunology at the Good Samaritan Hospital of Johns Hopkins University. In that location from 1976 to 1980 I had the fortunate opportunity to meet almost daily Kimi and Terry Ishizaka, to be able to enjoy their precious suggestions and their always constructive criticism and to establish a friendly relationship. In the following years, a number of Italian researchers from different Universities had the opportunity to meet the Ishizakas in Baltimore, to attend their seminars and to interact with their almost always extraordinary collaborators. Among these, it is sufficient to mention Tomio Tada, the discoverer of T suppressor lymphocytes and Tadamitsu Kishimoto, the discoverer of IL-6 and its receptor. Among the few non-Japanese I remember Thomas Platts-Mills, the discoverer of the Dermatophagoides antigens and a member of the Royal Society of London.

In 1989, Ishizakas moved to the West Coast and Kimi became first Scientific Director, later President and finally President Emeritus of La Jolla Institute for Allergy and Immunology, one of the most qualified research centers in the world in the field of Immunology.

The scientific production of Kimishige Ishizaka is monumental and of the highest level. He will be remembered among the Masters of Immunology for the discovery of IgE and for having elegantly elucidated their role in allergic reactions. For these discoveries, Kimi Ishizaka has received countless and prestigious scientific prizes including the Paul Ehrlich Award, the Emperor Award, the American College of Physicians Award and the First Order of Merit of the Sacred Treasure. Many distinguished Immunologists believe that he deserved to receive Stockholm’s honor for his discovery which revolutionized not only the pathophysiology, but also the immunological therapies of allergic diseases. The latest publication of Prof. Kimishige Ishizaka, entitled “The Way We Walked with Immunology” (Annu. Rev. Immunol 36: 1, 2018), summarizes his extraordinary scientific journey that places him among the giants of modern Immunology.

Prof. Ishizaka has been President of prestigious Scientific Societies such as the American Academy of Allergy and Clinical Immunology, the American Society of Immunology and the *Collegium Internationale Allergologicum*. In this role, he honored our country by choosing Sorrento (October 1982) as the site of the X *Collegium Internationale Allergologicum*, which I had the honor of organizing, under his friendly supervision, together with the late Professors Mario Condorelli and Mario Ricci.

Gianni Marone

Past-President

*Collegium Internationale Allergologicum*

